# Factors Associated with Anxiety and Depression in Infertile Couples—Study Protocol

**DOI:** 10.3390/healthcare10071352

**Published:** 2022-07-21

**Authors:** Tong Yang, Nahathai Wongpakaran, Tinakon Wongpakaran, Ubol Saeng-Anan, Charuk Singhapreecha, Rewadee Jenraumjit, Carmelle Peisah

**Affiliations:** 1 Master of Science (Mental Health), Graduate School, Chiang Mai University, Chiang Mai 50200, Thailand; tong_yang@cmu.ac.th (T.Y.); tinakon.w@cmu.ac.th (T.W.); charuk.s@cmu.ac.th (C.S.); rewadee.w@cmu.ac.th (R.J.); carmelle.peisah@health.nsw.gov.au (C.P.); 2 Department of Psychiatry, Faculty of Medicine, Chiang Mai University, Chiang Mai 50200, Thailand; 3 Division of Reproductive Medicine, Department of Obstetrics and Gynecology, Faculty of Medicine, Chiang Mai University, Chiang Mai 50200, Thailand; ubol.saeng@cmu.ac.th; 4 Faculty of Economics, Chiang Mai University, Chiang Mai 50200, Thailand; 5 Department of Pharmaceutical Care, Faculty of Pharmacy, Chiang Mai University, Chiang Mai 50200, Thailand; 6 Discipline of Psychiatry and Mental Health, Faculty of Medicine, University of New South Wales, Sydney, NSW 2052, Australia; 7 Specialty of Psychiatry, Faculty of Medicine and Health, University of Sydney, Sydney, NSW 2006, Australia

**Keywords:** anxiety, depression, infertility, mental health, couple, dyadic analysis, actor-partner interdependence model

## Abstract

(1) Background: Infertility refers to the failure to achieve a pregnancy after 12 months or more of regular unprotected sexual intercourse. Infertility is an important medical and social problem that causes individual distress, family conflict and emotional impact experienced by about 15% of couples worldwide. Anxiety and depression are the main psychological problems associated with infertility with many potential contributing factors which are yet to be fully elucidated. This study aims to investigate factors related to anxiety and depression among infertile couples. (2) Methods/Design: This study will employ an analytical cross-sectional survey. Sociodemographic information will be collected. Validated tools will be used to assess anxiety and depression (Outcome Inventory-21(OI-21), marital satisfaction (ENRICH Marital Satisfaction Scale, sufficiency economy (Sufficiency Economy Scale (SES) and personality traits (Zuckerman-Kuhlman-Aluja Personality Questionnaire (ZKA-PQ). The Actor-Partner Interdependence Model estimated by multilevel modeling will be used for dyadic analysis. (3) Discussion: This study will provide evidence about factors associated with anxiety and depression in infertile couples. Outcomes will raise awareness about mental health problems among infertile couples and guide future research for interventions.

## 1. Introduction

Infertility refers to the failure to achieve a pregnancy after 12 months or more of regular unprotected sexual intercourse [1,2]. Infertility affects millions of people around the world, including the couple themselves and extending to their families and wider communities. Moreover, contrary to popular misperception, this is a problem shared by men and women alike. Many couples who want to get pregnant but struggle with infertility attribute the infertility to the female partner. Nevertheless, of all infertility cases, approximately 20–30% are related to both male factors and female factors. The female factor is implicated in about 50% cases of infertility. At least 30% of infertility cases are identified as male causes [3,4,5]. About 85% of infertility cases can be identified. The rest of the infertility cases are still unexplained [6].

Infertility is an important medical and social problem that causes individual distress, family conflict and emotional impact on couples. Discrimination, social exclusion and abandonment can bring about significant mental health distress amongst infertile couples in pronatalist cultures such as Asia. Furthermore, the prevalence of infertility is increasing in both developing and developed countries [7]. For over three decades, due to techno-logical advances, an increasing number of infertile couples have been made aware of and are seeking infertility treatment, some with successful outcomes, although not all [8].

Anxiety and depression are considered the main mental health disorders associated with infertility, with a female preponderance for anxiety [9,10]. Several studies report adverse mental health outcomes, in particular depression and anxiety, associated with infertility per se and infertility treatment. Infertile couples experienced failure of infertility treatment [11], low income and polygamy [12] have higher prevalence of anxiety and depression. Moreover, there is evidence of a link between gender and anxiety/depression in infertile couples. A cross-sectional study in infertile patients in Iran showed that women were 2.54 times more likely than men to suffer from anxiety [9]. Similarly, a recent study Turkey investigated anxiety and depression in couples undergoing assisted reproduction treatment, found the incidence of depressive and/or anxious symptoms in women higher than in men [13]. Parents who gave birth to twins after infertility treatment were more likely to have higher level of anxiety and depression than parents who had only one child, according to the results of a prospective longitudinal study of couples who had been successfully treated for infertility [10].The urgent need for detecting and caring for these disorders is because they have impact on quality of life as well as they are associated with comorbidities such as substance use and suicidality. Moreover, it is known that anxiety and depression in infertile couples affects adherence to infertility treatment and follow-up [11]. It is important to raise awareness of the mental health of infertile couples and more research is needed to explore the unknown factors associated with anxiety and depression in infertile couples in Thailand or in Asia.

Known and potential factors influencing mental health outcomes in infertile couples

Repeated fertility treatment

A survey of infertile couples who had failed repeated fertility treatments and those who had not received any treatment, compared with couples with no history of infertility who had at least one child, showed that infertile couples had lower quality of life [14]. Among infertility patients undergoing infertility treatment, the number of infertility treatments was associated with prenatal anxiety and depression [15]. Finally, infertile women reported higher levels of anxiety in the event of infertility treatment failure and prolonged infertility treatment [16].

Personality

Personality is a known determinant of mental health outcomes, with studies showing that neuroticism (which reflects negative emotions and emotional instability) is negatively correlated with inner strength [17] and is a risk factor for both depression [18] and anxiety [19]. More specifically, one study has found an association between certain personality traits and depression in infertile women [20]. However, there is still a lack of research exploring the relationship between personality and anxiety and depression in infertile couples, an important gap that needs to be addressed.

Substance use disorders

There is evidence that alcohol addiction is a risk factor for generalized anxiety disorder, social phobia and panic disorder [21]. Daily cannabis use or dependency disorder can lead to anxiety and when comorbid, the management of both anxiety and substance abuse disorders must be addressed [22].

Moreover, persistent use of multiple substances is associated with depression. Men, in particular, have higher rates of depression [23]. There is evidence that substance abuse increases the risk of suicide in people with depression [24]. Early substance use has been associated with depression, including early alcohol use and early use of marijuana and other illegal drugs [25]. Little is known about substance abuse and mental health outcomes of infertility.

Income

Despite the fact that there is a gradual increase of monthly income per household in Thailand [26], the role of income remains influential on mental health outcomes of family members. A study in Thailand showed that people with higher income and better economic status had a lower risk of mental illness [27]. Therefore, it is reasonable to investigate whether income is a related factor for anxiety and depression in infertile couples in Thailand.

Sufficiency Economy concept

Sufficiency economy is a philosophy of life and governance based on Thailand’s national conditions that can be applied to individuals, families and communities at all levels. Sufficiency economy has been widely used in the past decade to guide Thai policy and all aspects of Thai people’s life [28]. The theory instructs people to value politics, honesty, diligence, knowledge, wisdom and sensitivity. Sufficiency economy contains three basic principles, namely moderation, reasonableness and inherent immunity. Moderation means moderation in all actions. Reasonableness demonstrates that the consequences of actions affect not only oneself but also others, society and the environment. Immunity is the ability to resist risk. It guides people to have a clear understanding of their own behavior, be honest and diligent in dealing with people, abide by the mean, make rational decisions, and build the ability to rise above risk. Sufficiency economy emphasizes a more balanced approach to life. It is a reminder that economic development is not an end goal, but a way to achieve it. If people fully understand the economy of well-being, they can increase life satisfaction and achieve more happiness [29]. This theory has had a significant impact on Thais’ lifestyle and is also closely related to mental health which may affect anxiety or depression in infertile couples.

Couples or intimate relationships

There is a two-way relationship between mental health and intimate (marital) relationships, with adverse mental health affecting marital outcomes and vice versa [30]. Good marital relationships have a positive impact on family members. The quality of the marital relationship affects not only the individual but also their social life [31].

The Actor–Partner Interdependence Model (APIM) is widely used for dyadic data analysis in interpersonal relationships [32]. Because marital satisfaction can affect the mental health of infertile couples [31], it is worth exploring whether one partner’s marital satisfaction has an effect on the other partner’s anxiety and depression to varying degrees.

### Current Study

The aim of the current study is to investigate the relationship between these independent variables (i.e., personality traits, marital relationships, sufficiency economy, demographic factors) and the dependent (outcome) variables of anxiety/depression in infertile couples.

Based on the reviews mentioned above, we hypothesize that

**Figure 1:**
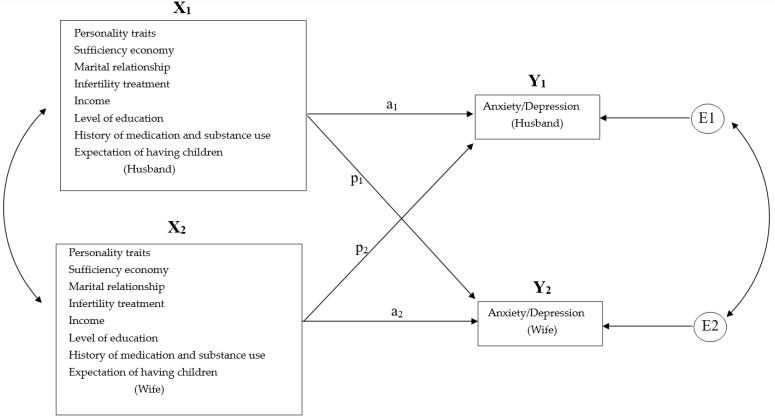
Actor–partner interdependence model (APIM) shows the actor effect, partner effect and interaction effect in infertile couples, e.g., X1: marital satisfaction; X2: wives’ marital satisfaction; Y1: husbands’ anxiety/depression; Y2: wives’ anxiety/depression; E1 & E2: corresponding error terms.

(i) personality traits, marital satisfaction, concept of sufficiency economy, infertility treatment, demographic factors and personal history (e.g., gender, age, expectation of having children, history of medication and substance use, income and level of education) are associated with anxiety and depression in infertile couples. More details can be found in Figure 1.
(ii) the interaction between spouses is linked to anxiety and depression in infertile couples. Specifically, there are three effects as we show in Figure 1. (1) Actor effect: participants’ independent variables are correlated with their anxiety or depression. (2) Partner effect: Participants’ independent variables are correlated with anxiety and depression in their spouses. (3) Interaction effect: Individuals’ independent variables and their spouses’ independent variables interact significantly. In other words, one partner’s independent variables affect the other’s anxiety and depression but only at certain levels.

## 2. Materials and Methods

### 2.1. Study Design and Time-Period

The study will employ a descriptive and analytic cross-sectional design among infertile couples attending the infertility centers under the Faculty of Medicine, Chiang Mai University and Chiang Mai IVF (In Vitro Fertilization) Clinic (June–September 2022). There will be one-time paper or digital questionnaire until the expected number is achieved.

### 2.2. Study Population

The participants will be infertile couples in Thailand. Inclusion criteria include couples (1) At least one person of the couple is diagnosed with infertility (primary/secondary) under ICD-10 and attends the infertility centers under the Faculty of Medicine, Chiang Mai University and Chiang Mai IVF Clinic, (2) Fluent in speaking, reading, and writing in Thai or English, (3) Accessing service either in pretreatment or undergoing treatment and (4) Consent to participate in research by oneself.

### 2.3. Procedure and Participant Invitation

First, the study will be approved by the Ethics Committee of Chiang Mai University. All tools/questionnaires will have both English version and Thai versions with good Cronbach’s alpha or the internal consistency of 0.80 at least. The investigators will invite infertile couples at the time of attendance at the infertility centers under the Faculty of Medicine, Chiang Mai University and Chiang Mai IVF Clinic to participate in the study. Alternatively, participants can also fill out questionnaires online. At least one of the couple must be conversant in English or Thai to participate in the study. We will distribute the questionnaire with the participants’ eligibility, information sheet (PIS) and the informed consent form (ICF) to infertile couples. Participants can withdraw at any time during the questionnaire answering process. They can ask any question about the questionnaire. This is an anonymous questionnaire. Participants’ information will be kept strictly confidential. The duration of data collection will be 3 months until we get adequate sample size. We will screen out valid questionnaires and filter out invalid ones. Finally, we will input data and analysis results.

### 2.4. Measurements

Participants will complete the questionnaire independently. We will have either the Thai or English versions available for the participants to choose which one they prefer. Moreover, according to the COVID-19 procedures, we will have both the paper and digital versions available for the participants’ preferences. The measurements include (1) Demographic data questionnaire (Table 1): It includes gender, age, income, educational level, medical history (substance abuse), family history of infertility, expectation of having children, treatment conditions, etc. (2) Stage and nature of infertility treatment will also be collected. Outcome Inventory-21(OI-21): It was used to measure the level of anxiety, depression, somatization and interpersonal difficulty [33]. (3) Zuckerman-Kuhlman-Aluja Personality Questionnaire (ZKA-PQ): In general, the five-factor model measures can show associations between predictors and personality traits include neuroticism (NE), sensation seeking (SS), extraversion (EX), activity (AC) and aggressiveness (AG) [34]. (4) ENRICH (Evaluation and Nurturing Relationship Issues, Communication and Happiness) Marital Satisfaction Scale is used to judge marital satisfaction and to identify causes of marital conflict [35]. (5) Sufficiency Economy Scale (SES): measuring sufficiency economy level. Generally, a higher score means better level of Sufficiency Economy [36]. We have all the measurements tested for their internal consistency in 20 Thai speaking adults and 43 English speaking adults and yield Cronbach’s alpha of at least 0.7. More details can be found in Table 2.

### 2.5. Statistical Analysis Plan

#### 2.5.1. Sample Size Calculation

We will use the number of predictors to estimate the sample size. G*Power will be used to calculate the sample size. We will use A Priori analysis to compute the required sample size under F tests. The effect size f2 is equal to 0.15. The α err prob is equal to 0.05. The power (1–err prob) is equal to 0.80. The number of predictors is equal to 11. As a result, we can get a total sample size of 123 couples. We estimate that there have been approximately 100 couples who have sought infertility treatment per month during the pandemic in the infertility centers under Faculty of Medicine, Chiang Mai University. In order to prevent incomplete data, we will add 20% to the estimated sample size. Therefore, we adjusted the sample size to 150 couples. We will give 50 THB per participant as compensation for their time volunteering for this research.

Sampling

We will undertake simple random sampling method [37] and try to invite every consecutive case on every-other-day fashion in two centers. The research assistants will invite participants to answer the questionnaire at the Fertility Center, Center for Medical Excellence (CMEx) on Monday, Wednesday and Friday. They will invite participants to answer the questionnaire at Chiang Mai IVF clinic on Tuesday, Thursday and Weekends. This simple random on the data collection will be for preventing the selection bias.

#### 2.5.2. Statistical Analysis

Descriptive analysis will be applied with demographic data (e.g., age, sex, education, income) infertility data (e.g., types of treatment), and scores of mental health factor (e.g., OI-21, ZKA-PQ, ENRICH Scale and SES scores) and will be presented by frequency, percentage mean, and standard deviation.

Differences among groups will be investigated by using the χ_2_ test or *t*-test according to whether the variables are categorical or continuous, e.g., intimate relationships with different levels of anxiety and depression. ANOVA will be used to check the differences among multiple groups, e.g., depression/anxiety total scores among different groups of income/education. Relationships among variables will be studied by using correlation statistics. Pearson’s correlation will be used to check the correlation between continuous variables (e.g., depression score and self-sufficiency economy) that are normally distributed. Spearman’s rank coefficient will be applied for testing correlation between two variables that are categorical and or ordinal such as level of anxiety and level of self-sufficiency economy.

The web-based interactive tool for analysis of the actor-partner interdependence model using multilevel modeling, written by David A. Kenny will be used in this study [36]. This modeling is as follows.

We will use error-dependent generalized least squares analysis and limited maximum likelihood estimation. Coefficient analysis used Z tests, and correlation determination using correlation coefficient *t* tests. The effect sizes of the actor and partner effects were partially correlated and ‘d’ when the predictor was dichotomous. Betas were employed twice, once using the population standard deviation for all (o) and once using the standard deviation for each parent (s). Beta (o) values were checked to compare equivalence between members, and all predictors were grand-mean-centered before the analysis. The partial correlations between predictor and outcome variables, controlling for all other predictors, were calculated as effect sizes. Values above r = 0.10 indicated a small effect size, between r = 0.30 and r = 0.50 a medium effect size and above r = 0.50, a large effect size [38]. Bonferroni correlation was performed to test multiple categories, e.g., ANOVA and chi-square test. In addition, the APIM was used. In APIM analysis, the different patterns of interdependence were tested using k parameters (a ratio of the partner effect on the actor effect) [39]. The four patterns are: (1) actor-only pattern, when a k parameter with a value is near 0, and a ≠ 0, *p* = 0, 2) partner-only pattern, when a k parameter with a value is near 0, and a = 0, *p* ≠ 0, 3) couple-oriented pattern, when a k parameter is near 1, and a = *p* and finally 4) contrast pattern, when a k parameter is near −1, and (a + *p* = 0) [40].

A *p*-value < 0.05 and 95% confidence interval will be considered significant. SPSS version 22 will be used to analyze the data.

### 2.6. Ethics Approval, Consent to Participate, Autonomy and Confidentiality

The study was approved by the ethics committee of the Faculty of Medicine, Chiang Mai University. We will obtain informed consent form the participants. It will be voluntary participation. Participants know the purpose, benefits and risks behind the study before they agree or decline to join. Participants’ information will be kept strictly confidential. Any personal information that they do not wish to disclose will be kept confidential. Their consent document will be kept separate from their questionnaire information to protect their identity. Only the researcher can access the results of participant’s questionnaire.

We will close the online platform and stop distribute the paper version after meeting the expected number. Only researchers can download the data. The research team will input the data to an offline version every few days. The information will be kept until data cleaning is complete and for about 1 year later. Then, the database and the account used to create it will be deleted permanently. During this time, personal information i.e., name (in ICF) will be protected and deleted as soon as possible. All the personal information will be kept offline and separated from the main database. Offline database will be kept in an anonymous form 5 years after the last publication. The researchers will delete it in the recycle bin.

## 3. Discussion

While we understand that women, regardless of culture, are more vulnerable to anxiety and depression, our understanding of other associations between infertility and mental health remain unclear. Previous studies on personality of infertile patients often focused on infertile women, not on infertile men. And no study involved both members of the infertile couple. Moreover, understanding the effect of infertility in pronatalist cultures such as Thai culture are important pieces in the puzzle to support infertile couples.

As far as we are aware, this is the first such study of both members of the couple seeking infertility treatment, and the most comprehensive examination of the multiple potential factors associated with anxiety and depression in infertile couples, in Thailand and internationally. Specifically, this is the first research to explore the relationship between personality traits, the sufficiency economy concept, infertility treatment, and other related factors (age, genetic history, expectation of having children, etc.) and anxiety/depression in infertile couples.

Research implication

The results will aid clinicians in their clinical practice by detecting depression and anxiety among patients while getting infertility treatment. The findings of associated factors to depression and anxiety will enable further prevention and intervention.

Limitation

We will invite infertile couples who seek treatment. Response and recall biases might occur because the data will be collected with a self-administered questionnaire. The researchers will consider social desirability bias that could occur as the participants are couples. We will interpret the data with caution. The fact that we have not included questions about polygamy, religion and whether the couples have extramarital relationships. The cross-sectional nature of the research limits causal relationships of the outcome.

## 4. Conclusions

This research will report the prevalence of anxiety and depression among infertile couples; and the relationship between these outcomes and many independent variables, including personality traits, marital relationships, and sufficiency economy. Findings from our study may benefit clinicians in identifying a case with such risk factors for anxiety and depression.

## Figures and Tables

**Table 1:** Demographic information.

Demographic	Choices
Sex	Male
	Female
Age	>20 years old
Occupation	Freelance
	Government or state enterprise
	Self-employed
	Unemployed
	Other
Educational level	Illiterate
	Primary school
	High school
	Vocational school
	Bachelor’s degree
	Higher
Monthly income (Baht)	0–25,000
	25,001–50,000
	50,001–75,000
	75,001–100,000
	100,001 or higher

**Table 2:** Measurement tools of the survey.

Instrument	Aim in Assessing	Response Format	Number of Items	Recall Period	Internal Consistency (Cronbach’s Alpha)
Outcome Inventory-21(OI-21)	Level of anxiety Level of depression Level of somatization Level of interpersonal difficulty	5	21	Past 1 week	English version: 0.873 Thai version: 0.937
Zuckerman-Kuhlman-Aluja Personality Questionnaire (ZKA-PQ)	Personality traits	4	40	Current	English version: 0.783 Thai version: 0.753
ENRICH Marital Satisfaction Scale	Marital satisfaction	5	15	Current	English version: 0.840 Thai version: 0.930
Sufficiency Economy Scale (SES)	Level of sufficiency economy	7	9	Current	English version: 0.70 Thai version: 0.75
